# Dewatering Behavior of a Wood-Cellulose Nanofibril Particulate System

**DOI:** 10.1038/s41598-019-51177-x

**Published:** 2019-10-10

**Authors:** Ezatollah (Nima) Amini, Mehdi Tajvidi, Douglas W. Bousfield, Douglas J. Gardner, Stephen M. Shaler

**Affiliations:** 1 School of Forest Resources and Advanced Structures and Composites Center, University of Maine, Orono, ME 04469 USA; 2 Department of Chemical and Biomedical Engineering, University of Maine, Orono, ME 04469 USA

**Keywords:** Materials science, Nanoscience and technology

## Abstract

The novel use of aqueous suspensions of cellulose nanofibrils (CNF) as an adhesive/binder in lignocellulosic-based composite manufacture requires the removal of a considerable amount of water from the furnish during processing, necessitating thorough understanding of the dewatering behavior referred to as “contact dewatering”. The dewatering behavior of a wood-CNF particulate system (wet furnish) was studied through pressure filtration tests, centrifugation, and characterization of hard-to-remove (HR) water, i.e. moisture content in the wet furnish at the transition between constant rate part and the falling rate part of evaporative change in mass from an isothermal thermogravimetric analysis (TGA). The effect of wood particle size thereby particle specific surface area on the dewatering performance of wet furnish was investigated. Permeability coefficients of wet furnish during pressure filtration experiments were also determined based on Darcy’s law for volumetric flow through a porous medium. Results revealed that specific particle surface area has a significant effect on the dewatering of wet furnish where dewatering rate significantly increased at higher specific particle surface area levels. While the permeability of the systems decreased over time in almost all cases, the most significant portion of dewatering occurred at very early stages of dewatering (less than 200 seconds) leading to a considerable increase in instantaneous dewatering when CNF particles come in contact with wood particles.

## Introduction

Cellulose nanofibrils (CNF) have received a tremendous level of attention over the past few years as potential binders, reinforcing fillers, paper coatings, oxygen barrier films, and filaments attributable to the unprecedented specific strength of the individual nanofibrils, low density, superb adhesion properties, chemically tunable surface functionality, renewability, and biological abundance of a material obtained from sustainable resources. Finding novel applications which can highly benefit from outstanding intrinsic properties of CNF has been the subject of numerous recent studies^1–9^.

CNF consists of nano and micro-scale cellulosic fibers suspended in water and is mostly available in the form of a low-consistency (less than 5 wt.%) aqueous suspension. It offers excellent adhesion properties attributed to a very high specific surface area and a vast number of hydroxyl groups available on the cellulosic surfaces, which make this type of material a superior candidate for many different applications^1,10^. The utilization of CNF as well as lignin-containing CNF (LCNF) as binders in the formulation of particleboards and medium density fiberboards has been reported^2,11–15^. Potential applications of CNF as a binder for the production of laminated papers^16^, reinforcing natural fiber yarns^17^, and self-assembly processes^3,18^ have been recently proposed.

The current processing technology to produce composite panels using CNF or LCNF as binder consists of a dewatering process followed by drying in a hot press^2,13–15^. To shorten press cycles and save energy, the majority of the water present in the mixture of wood particles and CNF (hereafter “furnish” or “mattress”) must be mechanically removed prior to hot pressing in an efficient manner. Therefore, understanding and controlling the water removal behavior of the CNF suspension, both solely and in the form of a mix with other materials is a critical step to optimize the production process.

The terms “dewatering” and “drainage”, herein, refer to liquid (assuming only water) removal from the solid-liquid mixtures during a filtration process. The material structure forming as dewatering progresses is referred to as “filter cake”. To date, the dewatering behavior of cellulosic suspensions and furnishes has been studied by many researchers mostly through filtration or rheological theories or combination of the two^19–29^. Paradis *et al*. used a modified dewatering apparatus equipped with a cone-and-plate rheometer to determine the drainage resistance coefficient of different grades of paper-making stock under a known shear condition. The influence of shear rate on the drainage resistance was also investigated, which pointed out that the drainage rate changes as a result of the change in the characteristics of the filter cake as drainage progresses^19^. Dimic-Misic *et al*. studied the effect of shear stress as well as swelling (expressed as the water retention value at a relatively low consistency) of micro and nanofibrillated cellulose (MNFC) on the dewatering behavior of the cellulose furnishes. It was found that the nanofibrillar suspension added to the pulp-pigment particles furnish predominantly governs the rheological and dewatering responses. Highly swelled nanofibrillated cellulose was shown to have a significantly difficult dewatering owing to plugging the bottom layer of the filter cakes with ultrafine fibrils. A noticeable gel-like structure as well as shear-thinning behavior –i.e. the decrease in viscosity under increasing shear rates– were seen for all the MNFC suspensions and furnishes, thus more efficient dewatering at higher shear rates could be attained^20,21^.

The influence of CNF flocculation upon charge neutralization by the addition of salt on the dewatering ability of CNF suspension was investigated using a pressure dewatering method and it was determined that the dewatering ability of the CNF suspension is affected by the type and concentration of the salt^22^. Rantanen *et al*. studied the effect of adding MNFC to the formulation of high filler content composite paper in the web dewatering process using a gravimetric dewatering evaluation. The results revealed that increasing the MNFC fibrillation decreased the dewatering performance, however, this could be tuned by *in situ* precipitation of precipitated calcium carbonate (PCC) to achieve a desirable combination of strength and processing performance^23^. Further assessments have been done to enhance the dewatering capability of MNFC suspensions and furnishes under an ultra-low shear rate (approx. 0.01 s^−1^), including the addition of colloidally unstable mineral particles (such as undispersed calcium carbonate), acid dissociation of the surface water bound to the nanofibrils of cellulose by adding ultrafine calcium carbonate nanoparticles, and controlling the rheological properties with respect to length and aspect ratio of fibrils^24–26^.

Clayton *et al*. studied the dewatering mechanisms of a range of biomaterials, including lignite, bio-solids, and bagasse, through mechanical thermal expression (MTE) using a compression-permeability cell. It was revealed that at lower temperatures the predominant dewatering mechanism is mechanical dewatering referred to as “consolidation” by the authors. However, thermal dewatering plays a more important role at higher temperatures^27^. A dynamic model was developed by Rainey *et al*. to predict the filtration behavior of bagasse pulp incorporating steady state compressibility and permeability parameters obtained from experimental data^28^. Hakovirta *et al*. employed a method to improve the dewatering efficiency of pulp furnish through the addition of hydrophobic fibers and demonstrated that adding a low percentage of hydrophobic fibers to the pulp furnish could impact freeness and water retention properties, thus a considerable improvement in the dewatering efficiency was attained^29^. A method was used to measure the permeability of fiber mats at different flow rates during the medium density fiberboard manufacturing process using Darcy’s law^30^. Lavrykova-Marrain and Ramarao employed two mathematical models based on conventional cake filtration theory and multiphase flow theory by applying Darcy’s law to describe dewatering of pulp fiber suspensions under varying pressure^31^. A model was also developed to predict the permeability of cellulose fibers in pulp and paper structures based on Kozeny-Carman theory assuming fibers are either cylindrical or band-shape in a two-dimensional network^32^. Darcy’s law was also applied to predict the weight of CNF-containing paper coatings through filtration theory^33^.

The original hypothesis of this study is based on the fact that in a CNF suspension, water is mostly in the form of adsorbed water associated with the cellulose surface and is tightly bound to the hydroxyl groups present in the amorphous regions through hydrogen bonding. After mixing wood particles (WPs) with CNF slurry, a large portion of the adsorbed water turns into free water as a result of contact between nanofibrils of cellulose and WPs, a phenomenon termed here as ‘contact dewatering’ first reported by our research group^1,2^. Upon consolidation, a considerable amount of free water is removed from the wet furnish by pressing (mechanical dewatering) in a very short period of time and the remaining water in the system can be removed through heating (evaporative dewatering) to produce the final product.

In this study, the dewatering behavior of WP-CNF wet furnish was studied through pressure filtration tests and centrifugation. The effect of wood particle size and therefore particle specific surface area on the dewatering properties of wet furnish was investigated. A method based on Darcy’s law for volumetric flow through a porous medium was used to determine the permeability coefficients of wet furnish during filtration test. Characterization of hard-to-remove (HR) water in wet furnish was also carried out using high resolution isothermal thermogravimetric analysis (TGA) to evaluate thermal dewatering properties of the samples. The results of this study will be helpful in the design of processing equipment for the production of wet-formed CNF bonded composite panels.

## Materials and Methods

### Materials

Southern yellow pine wood particles (WP) with an average aspect ratio of 3.3 and average moisture content of 7% were supplied by Georgia-Pacific Thomson Particleboard (Thomson, GA, USA). The CNF was received in the form of a slurry of 3 wt.% cellulose nanofibrils from the University of Maine’s Process Development Center, which was the product of mechanical refining of bleached softwood kraft pulp. The properties of this CNF material are published elsewhere^3^. Polypropylene (PP) granules with the average diameter of 2.5 mm were provided by Channel Prime Alliance Inc. (Des Moines, IA, USA).

### Particle size distribution

To investigate the effect of WP size on the dewatering behavior of the wet furnish, particles were separated based on the size using a Retsch AS 200 laboratory sieve shaker (Retsch®, Haan, Germany). Particles were screened into six different size ranges, including larger than 2 mm (Group I), larger than 1.4 mm and smaller than 2 mm (Group II), larger than 1 mm and smaller than 1.4 mm (Group III), larger than 0.5 mm and smaller than 1 mm (Group IV), larger than 0.25 mm and smaller than 0.5 mm (Group V), and finally dust (Group VI).

**Figure 1:**
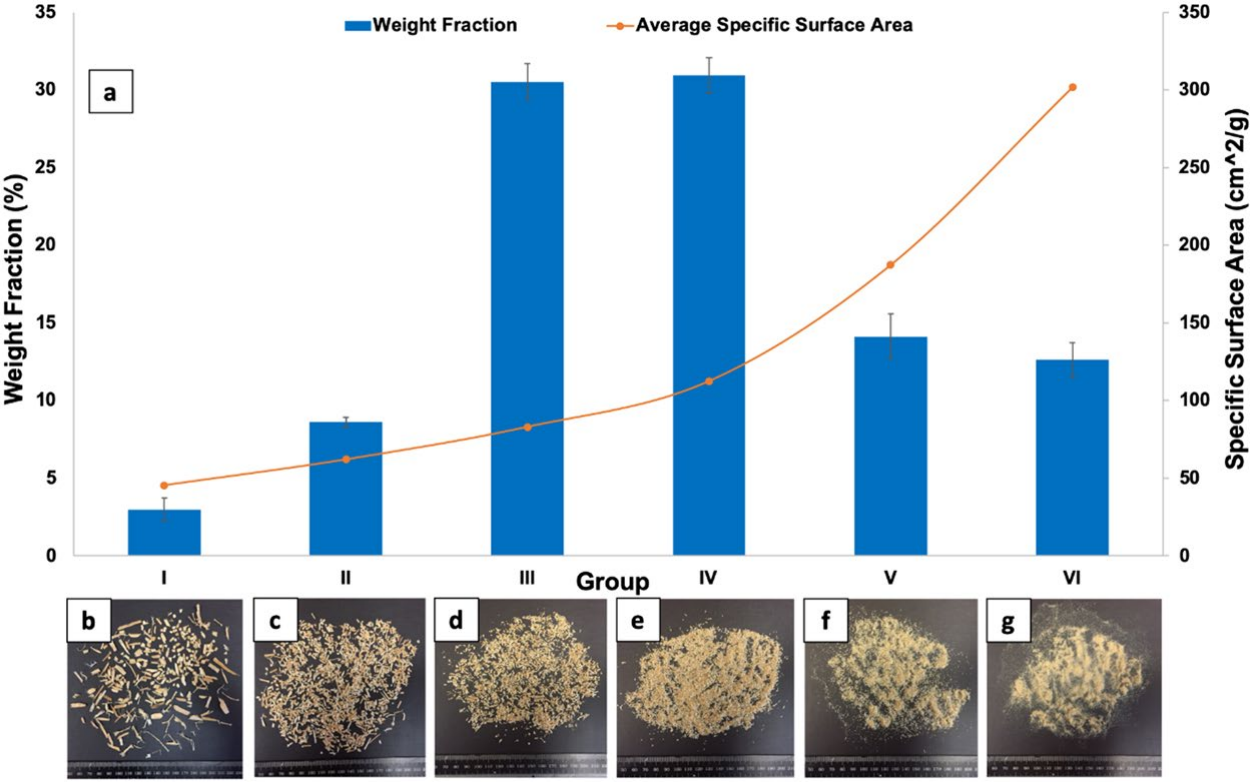
(**a**) WP size distribution and average specific surface area values in a given sample. WPs (**b**) larger than 2 mm (Group I) (**c**) larger than 1.4 mm and smaller than 2 mm (Group II) (**d**) larger than 1 mm and smaller than 1.4 mm (Group III) (**e**) larger than 0.5 mm and smaller than 1 mm (Group IV) (**f**) larger than 0.25 mm and smaller than 0.5 mm (Group V) (**g**) dust (Group VI).

The sieved particles were then weighed and the weight fractions of each particle size range was calculated based on the total weight of the given sample of WPs. Results are presented in Fig. 1. As shown in Fig. 1, WPs with the sizes ranging from 0.5 mm to 1.4 mm had the highest weight fraction, almost 60%, of the entire sample.

To determine the average specific surface area of the wood particles in each range/group, three different samples of wood particles, each sample about 5 grams in weight, were selected from each size range. The average thickness of particles in each sample was calculated through measuring the thicknesses of one hundred particles randomly selected from the given sample. The average length and surface area of each given sample were measured by an optical (digital) photograph of the sample and then processing the digital image using the ImageJ image processing software version 1.49 v (National Institutes of Health, USA). Assuming that particles are in the form of small cuboids and having the average values of length, thickness, and surface area, the average specific surface area of particles in a given sample can be approximated using Eq. 1:

1 $$SS{\rm{^{\prime} }}A=\frac{2\times [(S{\rm{^{\prime} }})+(a{\rm{^{\prime} }}+b^{\prime} )\times t{\rm{^{\prime} }}]}{w}$$

where $SS^{\prime} A$ is the average specific surface area (cm^2^/g), ($S^{\prime}$) is the average top view surface (cm^2^), $a^{\prime}$ is the average length (cm), $b^{\prime}$ is the average width (cm), $t^{\prime}$ is the average thickness, and w is the sample weight (g). The average width of the particles can be easily calculated by having the average top view surface and the average length through Eq. 2:

2 $$b^{\prime} =\frac{S^{\prime} }{a^{\prime} }$$

The average values of specific surface area for each particle size group are illustrated in Fig. 1. It is clearly shown that the smaller the wood particle size, the higher the specific surface area.

#### Pressure filtration

**Figure 2:**
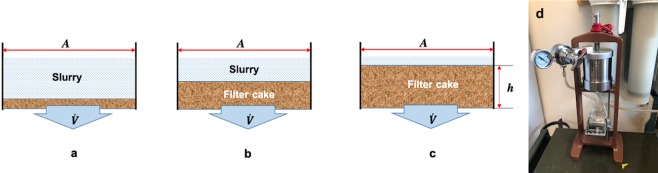
Schematic of the filtration model: (**a**) shortly after the beginning (**b**) in the middle (**c**) at the end of filtration experiment. (**d**) filter press and test setup.

A pressure filtration test was used as a method to study the dewatering behavior of the wet furnish. To investigate the effect of particle size on the dewatering of the wet furnish, samples of WPs with deferent sizes from Groups I through VI (excluding Group IV that had close SSA to Group III) were selected and mixed with a CNF slurry at 3 wt.% solids content. The mixing ratio of WPs to CNF was 7:3 based on dry weights of the constituents. Samples of pure CNF slurries with consistencies of 3 and 10 wt.% were also used to compare the dewatering behavior of pure CNF with that of WP-CNF mixes. The reason for choosing CNF 10 wt.% was that it had the same solids content as the mix samples. Pressure filtration tests were then carried out on the prepared samples at a pressure of 172 kPa (approx. 25 psi) for 30 minutes using an OFITE_®_ low pressure bench mount filter press (OFI Testing Equipment, Inc., Houston, TX, USA). Samples of 100 g from each formulation were loaded into the cylindrical chamber of the device on top of a metal screen and a filter paper. A small digital scale along with a glass Erlenmeyer flask on top were placed under the chamber outlet to collect and weigh the removing water (Fig. 2d). The changes in the weight of collected water through time were recorded by a video camera from which dewatering values were extracted.

#### Determination of permeability

Darcy’s law for liquid flow through a porous medium was used to determine the permeability of pure CNF and WP-CNF mixtures. A schematic of pressure filtration is illustrated in Fig. 2a–c. According to Darcy’s law, the specific volumetric flow rate ($\dot{V}$) is related to the pressure drop through the filter cake (∆***P***), permeability of the filter medium (***k***), viscosity of the fluid (*μ*) and the cake thickness (*h*):

3 $$\dot{V}=\frac{d(\frac{V}{A})}{dt}=\frac{\Delta Pk}{\mu h}$$

where $(\frac{V}{A})$ represents volumetric liquid flow per unit area and ***t*** is the drainage time. The thickness of filter cake (***h***) can be also obtained from Eq. 4 taking into account a balance between the volume of fibers trapped in the filter cake and the volume of fibers that were present in the water which has passed through the membrane at any given time^34^:

4 $$h=\frac{V{\varphi }_{0}}{A{\varphi }_{m}}$$

where *ϕ*_0_ and *ϕ*_*m*_ are volume fraction of fibers in the slurry and in the filter cake, respectively. In the case of wet furnish, fibers refer to the sum of cellulose nanofibrils and wood particles. The volume fraction of fibers can be easily obtained based on solids content of the slurry and the densities of fibers and water:

5 $$\varphi =\frac{s{\rho }_{w}}{s{\rho }_{w}+(1-s){\rho }_{f}}$$

where *s*, *ρ*_*w*_, and *ρ*_*f*_ are solids content of the slurry, density of water (for simplification assumed 1 g/cm^3^), and density of fibers, respectively. Rewriting Eq. 3 based on Eq. 4 will yield:

6 $$(\frac{V}{A})d(\frac{V}{A})=(\frac{\Delta Pk{\varphi }_{m}}{\mu {\varphi }_{0}})dt$$

Equation 7 can be derived from Eq. 6 by integration. Equation 7 actually describes the dewatering behavior based on the permeability and fiber volume fraction of the filter cake. This equation clearly demonstrates that the volumetric flow of water per unit area of the filter cake has a square root relationship with the pressure drop through the filter cake, permeability of the cake, volume fraction of fibers, and dewatering time.

7 $$\frac{V}{A}=\sqrt{\frac{2\Delta Pk{\varphi }_{m}t}{\mu {\varphi }_{0}}}$$

It should be noted that during the dewatering of wet furnish, the permeability of the filter cake changes due to the densification and compression of the filter cake over the time. To determine the permeability of wet furnish, Eq. 7 can be rearranged in the form of Eq. 8.

8 $$(\frac{\mu {\varphi }_{0}}{2\Delta P{\varphi }_{m}}){(\frac{V}{A})}^{2}=\kappa t$$

Volumetric liquid flow $(\frac{V}{A})$ can be calculated based on the filtrate mass (g) over filtration time (s), density of water (g/mm^3^), and cross-sectional area of the filter cake (mm^2^), which is roughly equivalent to the cross-sectional area of the cylindrical chamber, using the following equation:

9 $$\frac{V}{A}=\frac{{m}_{w}}{{\rho }_{w}A}$$

where *m*_*w*_ and *A* are mass of removed water and cross-sectional area of the filter cake, respectively. The left-hand side of Eq. 8 for each corresponding volumetric flow can be calculated and plotted versus time. The permeability of filter cake at each time interval can then be determined by fitting a straight line to the resultant curve in the corresponding time interval and finding the slope of the lines.

#### Centrifugation

Water retention value (WRV) of wet furnish gives a useful measure of the performance of fibers and particles relative to the dewatering behavior of the furnish. Samples of WP-CNF mixtures along with pure CNF 3 wt.% and 10 wt.% were prepared using the same preparation method as the pressure filtration experiment. Samples of WP Group I were excluded from the experiment owing to insufficiency. The WRVs of the samples were determined through centrifugation at 2200 rpm for 15 minutes using a CLAY ADAMS DYNAC^®^ II table top centrifuge (Becton, Dickinson and Company, Franklin Lakes, NJ, USA). In order to separate the water removed during the centrifugation from the wet furnish and collect the leftover furnish, a Pierce^TM^ Protein Concentrator PES tube was used. A round piece of filter paper was cut out of the filter paper used for the pressure filtration test and placed underneath the samples prior to centrifugation to have control over the liquid flow and not to clog the tube membrane. After centrifugation, the leftover furnish was removed and weighed to determine the weight of centrifuged furnish. Samples then were dried in an oven at 105 °C until they reached the constant weights. The water retention values were calculated using Eq. 10.

10 $$\mathrm{WRV} \% =\frac{{W}_{w}-{W}_{d}}{{W}_{d}}\times 100$$

where *W*_*w*_ and *W*_*d*_ are the wet weight of the sample after centrifugation and the oven-dry weight of the sample, respectively.

#### Hard-to-remove water

**Figure 3:**
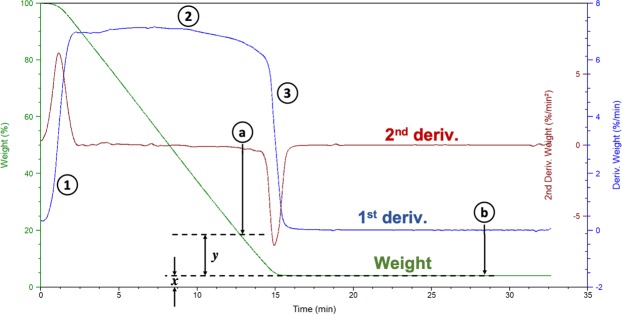
Representation of drying response during an isothermal heating protocol used to define hard-to-remove water.

Evaporative dewatering is another important mechanism of water removal occurring during the hot pressing process. To investigate the influence of particle size on the evaporative dewatering of the wet furnish, high resolution isothermal thermogravimetric analysis (TGA) was used based on the method first proposed by Park *et al*.^35^ for measuring what was termed “hard-to-remove (HR) water”, in softwood bleached kraft pulp fibers. HR water content is defined as the water content in fibers at the beginning of the transition between the constant rate zone and the dropping rate zone (between Part (2) and Part (3) in Fig. 3) of the evaporative change in mass (1^st^ derivative curve). It can be calculated by dividing the mass of water in the fiber associated with the starting point (Point (a) in Fig. 3) of the transition stage by the mass of the dried fiber (Point (b) in Fig. 3), i.e. *y* divided by *x* in Fig. 3. To find the beginning of the transition stage, the starting point on the changes of the evaporative change in mass, i.e. 2^nd^ derivative curve is first located. Then the corresponding weight of water (value of “y”) at Point (a) from the TG curve is found. The HR water value can then be calculated by simply dividing the obtained “y” value by the dry weight of the sample (value of x).

Samples of WPs from Groups III, IV, V, and VI were selected and mixed with a CNF slurry at 3 wt.% solids content. The mixing ratio of WPs to CNF was 7:3 on a dry-weight basis. Samples of pure CNF and pure WP slurries with the same solids content (3 wt.%) were also prepared and tested using a TGA (model Q500, TA Instruments, New Castle, DE, USA) with a heating regime of ramping up (100 °C/min) to 120 °C and then continuing isothermally at 120 °C for 30 minutes to assure that samples are fully dried. WPs with the size of larger than 1.4 mm -i.e. Group I and II- were excluded from the experiment due to the difficulty in filling the small TGA pans with relatively large WPs. To compare the HR water content of pure CNF with larger cellulosic fibers, samples of pure (3 wt.% consistency) softwood bleached kraft pulp were also tested. To investigate the effect of using a nonpolar and hydrophobic materials instead of WP in the formulation of the mix, samples of 70% PP granules mixed with 30% CNF 3 wt.% (dry-basis) were made and tested as well. The initial mass of each sample was about 100 mg.

#### Statistical analysis

The experimental data were statistically analyzed using IBM SPSS Statistics Version 25 (IBM Corp., Armonk, NY, USA). A one-way ANOVA test was carried out to statistically compare the HR water properties as well as WRV results. Duncan’s multiple range test (DMRT) was also used to evaluate the group means. Comparisons were drawn based on a 95% confidence level.

## Results and Discussion

### Pressure filtration

**Figure 4:**
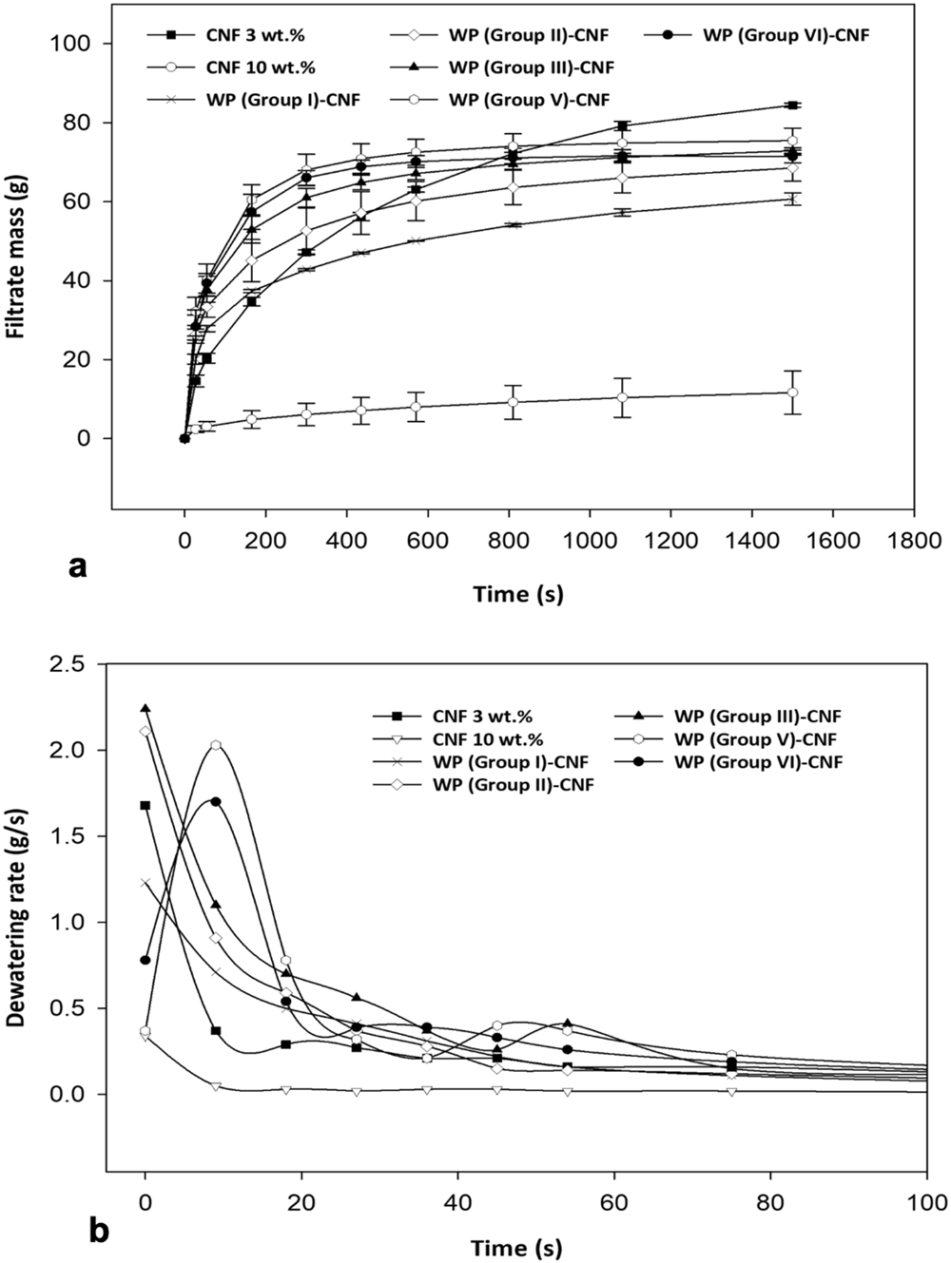
Average (**a**) water removal (**b**) dewatering rate over filtration time for various material formulations.

Pressure filtration tests revealed that the dewatering rate generally decreases over time, regardless of the material formulation. Samples of pure CNF 10 wt.% exhibited considerably lower amounts and rates of water removal within the same period of time compared to WP-CNF mixes with the same solids content (Fig. 4a,b). This also happened for the case of CNF 3 wt.% within the first 200 seconds of the filtration during which in other formulations most of the water removal occurred and dewatering rate started to level off. The dewatering rate of CNF 3 wt.%, however, continued to decrease until almost 20 minutes after the experiment started. This may support the original hypothesis that most of the water in a WP-CNF mix is in the form of free water owing to contact dewatering and could be easily removed from the system, however in pure CNF suspensions, adsorbed water predominantly exists, which is harder to drain. Higher levels of water removal at the end of the test in CNF 3 wt.% compared to other formulations may be related to the lower consistency of CNF 3 wt.% samples which was lower than all other formulations.

Among WP-CNF samples, those with smaller particle sizes- i.e. Groups V and VI- in general exhibited the highest levels of water removal during filtration experiments (Fig. 4a). This can be attributable to the smaller size, thus higher specific surface area, which resulted in higher levels of contact dewatering. The lowest level of dewatering (Fig. 4a) and smallest change in the rate of dewatering (Fig. 4b) occurred throughout the filtration of WP with the largest particle size and smallest specific surface area (Group I). This can be also explained by lower levels of contact dewatering in particles with smaller specific surface area. WP-CNF samples of Group V and VI showed to have a small amount of drainage even before applying any pressure. As shown in Fig. 4b, the initial increases in the dewatering rates of these two formulations within the first 10 seconds of the filtration is attributable to the pressure adjustments at the beginning of the experiments.

### Permeability

Permeability values of the samples were determined using Eq. 8. The values of $(\frac{V}{A})$ at each time were calculated through Eq. 9 by inserting the corresponding filtrate mass, density of water (1000 kg/m^3^), and the cross-sectional area of the cylindrical chamber (4.6 × 10^−3^ m^2^). The obtained volumetric flow values along with the pressure (172 kPa), viscosity of water (10^−3^ Pa.s) were then plugged into the Eq. 8. Initial volume fraction of fiber (*ϕ*_0_) and volume fraction of fiber at the end of the experiment (*ϕ*_*m*_) were also calculated through Eq. 5 and by measuring the solids of furnish before and after each filtration test.

Permeability values were obtained by plotting the left-hand side of the Eq. 8 over time and fitting a line to the resultant curve at certain time intervals. As for nearly all the formulations, the resultant curves corresponding to Eq. 8 showed three different regions with significantly different slopes- i.e. at the beginning, before reaching the plateau, and the plateau-, the permeability values for each formulation were determined over these three regions. Therefore, the obtained k_1_, k_2_, and k_3_ values respectively corresponded to the permeability of wet furnish at the beginning of the filtration, before reaching the point at which the dewatering rate started to level off, and at the level where no changes were seen in the dewatering rate. The obtained permeability values for each formulation are presented in Table 1. It can be seen that almost for all cases, the permeability decreases as the filtration goes on. The reduction in the permeability coefficient is more significant in WP (Group V and VI) mixtures with lower particle sizes. This can be attributed to the higher compaction and densification of smaller particles upon dewatering, which resulted in lower porosity in these materials.

**Table 1:** Average values of permeability over three regions and instantaneous dewatering.

Formulation	Permeability (m^2^)			Instantaneous dewatering (g)
	k_1_	k_2_	k_3_	
CNF 3 wt.%	10^−16^	7 × 10^−17^	1.7 × 10^−17^	3.3
CNF 10 wt.%	1.1 × 10^−17^	1.1 × 10^−17^	10^−17^	0.66
WP (Group I)-CNF	6.5 × 10^−16^	8.5 × 10^−17^	4.5 × 10^−17^	6.61
WP (Group II)-CNF	9.3 × 10^−16^	8.3 × 10^−17^	3.3 × 10^−17^	9.56
WP (Group III)-CNF	6.9 × 10^−16^	4 × 10^−16^	2.7 × 10^−16^	10.59
WP (Group V)-CNF	7.7 × 10^−16^	5.7 × 10^−17^	8 × 10^−18^	8.52
WP (Group VI)-CNF	8.3×10^−16^	2 × 10^−17^	2 × 10^−18^	9.55

Our observations in the lab and pressure filtration results indicated that the contact dewatering starts almost instantaneously after CNF particles come in contact with wood particles. To have a better understanding of how much water was instantaneously removed at the beginning of filtration, the instantaneous dewatering value for each formulation was obtained by plotting the logarithm of filtrate mass versus the logarithm of time and then fitting a straight line to the resultant curve. The intercept of the regression line yielded the logarithmic value of the instant dewatering. As presented in Table 1, CNF 10 wt.% has a significantly lower instantaneous dewatering value compared to that of the WP-CNF mixtures. The amount of the water immediately removed at the beginning of the filtration is even considerably lower in CNF 3 wt.%, as compared with that of the mixes. It clearly shows that, in general, adding WPs to CNF helps with the dewatering. For comparison the instantaneous dewatering of the 3 wt.% CNF increased by 100% when the largest wood particles were added to the system. This was increased by 220% when Group III wood particles were mixed with CNF.

### Water retention value

**Figure 5:**
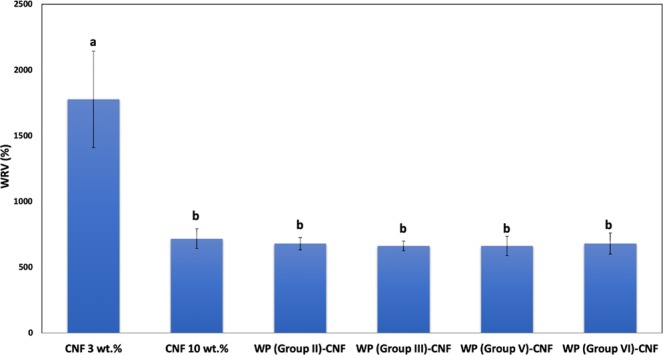
Average water retention values. Common letters over bars indicate no significant difference at 95% confidence level.

Water retention values of wet furnishes are shown in Fig. 5. Results simply showed that the level of final water removed is almost the same among CNF 10 wt.% and WP-CNF mixes with the same solids content. Higher level of water retention in CNF 3 wt.% shows that the percent ratio of water contained in the sample after centrifugation, within the same time and speed, is much higher compared to other formulations. As WRV test only measures the final amount of removed water, these tests cannot capture the change in the rate of dewatering unless tests are done for very short periods of time.

### Hard-to-remove water

**Figure 6:**
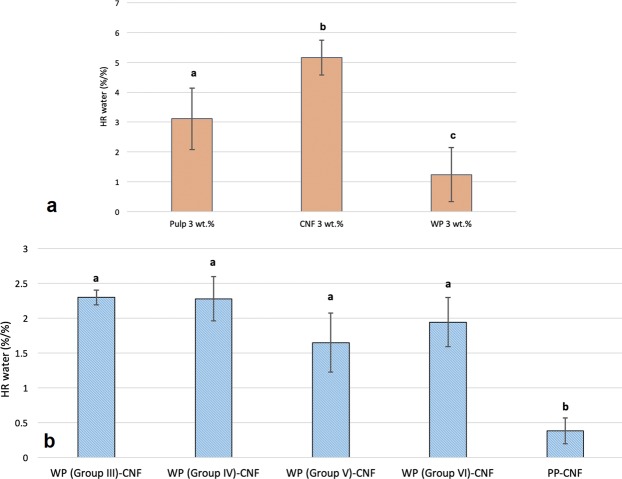
HR water values of (**a**) neat samples (**b**) mixed samples. Common letters over bars indicate no significant difference at 95% confidence level.

Results of HR water measurements are shown in Fig. 6. The HR water values of neat CNF samples were significantly higher than those of neat pulp and neat WP slurries with the same consistency. This can be interpreted as a higher amount of adsorbed water in the structure of CNF 3 wt.% slurry compared to pulp 3 wt.% and WP 3 wt.% suspensions as a result of much higher surface area and higher level of bound water in the fibrillar structure of the CNF. Moreover, WPs contain lignin, which is presumed to be less hydrophilic than neat CNF and pulp samples. Among the mixes, samples of PP-CNF showed the lowest levels of HR water attributable to the hydrophobicity and non-polarity of PP particles, most of the water in the system after mixing can be easily evaporated and can be considered free water. There were no significant changes observed among the HR water values of WPs (with different sizes) and CNF mixtures. This can be explained by taking into account the role of permeability on the one hand and the effect of particle size upon contact dewatering on the other hand. It was expected that smaller wood particles, because of having higher specific surface areas, should lead into higher amounts of contact dewatering. However, larger particles will cause easier evaporation owing to higher permeability. These two factors might have counter effects leading to no considerable difference in HR values.

In the work by Park *et al*., HR water content was measured in pulp fibers by determining the onset of transition between constant rate and falling rate zones through 2nd derivatives. The values found for softwood bleached kraft pulps were in the same range as our results, i.e. between 2 and 4 g/g. However, the solids content used in the study was not clearly mentioned. In another work, Sen *et al*.^36^ used another method to calculate the HR water for pulp fibers by integrating the area above the 1st derivative curve in the constant and falling rate zones, and compared this method with the method used by Park *et al*. The authors refined cellulose fibers to liberate microfibrils with different sizes ranging from several microns down to hundreds of nanometers. The values obtained for the microfibrillated cellulose are between 4 and 4.5 g/g, which were again in the same range as our results although again the solids used in their work were not clearly mentioned. Overall, although the results of HR water were useful for understanding the evaporative dewatering behavior of the wet furnish, the method did not seem to be capable of illustrating the effect of particle size on the contact dewatering clearly.

## Conclusions

Production of composite panels using CNF as an adhesive/binder is accompanied by a considerable level of water removal prior to hot pressing, which impacts pressing efficiency and energy consumption. This study focused on the dewatering behavior of WP-CNF particulate systems to understand and hence control the water removal from wet furnish. It was hypothesized that the size of WPs and consequently the specific surface area affects the level of contact dewatering, resulting from contact between nanofibrils of cellulose and WPs upon mixing. Pressure filtration tests were carried out to investigate the effect of particle size on the mechanical dewatering of wet furnish. It was found that among WP-CNF mixtures in general, those with smaller particle size had higher levels of water removal during filtration experiments. The lowest level of dewatering and smallest change in the drainage rate occurred during the filtration of WP with the largest particle size and smallest specific surface area (Group I). Samples of pure CNF 3 wt.% and 10 wt.% generally exhibited lower rates of water removal, as compared with those of WP-CNF mixes. This may support the original hypothesis that most of the water in a WP-CNF mix is in the form of free water as a result of contact dewatering and can be easily removed from the system, however in pure CNF suspensions, adsorbed water predominantly exists, which is harder to drain. The determination of the permeability coefficients of wet furnishes showed that regardless of the material formulation, the permeability of the wet furnish decreases over filtration time. The reduction in the permeability coefficients is more significant in WP mixtures with lower particle sizes (Group V and VI). This can be attributable to the higher compaction and densification of smaller particles upon dewatering that resulted in lower porosity.

Water retention values of wet furnish were measured through centrifugation technique. Results revealed that the amount of final water removed is almost the same among CNF 10 wt.% and WP-CNF mixes with the same solids content indicating that water retention values cannot capture the change in the rate of dewatering and therefore are unable to quantify contact dewatering. Samples of pure CNF 3 wt.% showed to have significantly higher level of water retention compared to other formulations, which simply means that the level of water contained in these samples after centrifugation under the same conditions is much higher compared to other formulations.

Characterization of HR water was also carried out to study the influence of particle size on the evaporative dewatering of wet furnish using high resolution isothermal thermogravimetric analysis (TGA). It was revealed that samples of neat CNF had higher values of HR water compared to neat pulp and neat WP suspensions with the same consistency. Samples of CNF mixed with PP showed the lowest levels of HR water attributed to the hydrophobicity and non-polarity of PP particles. Among the samples of CNF mixed with different sizes of WPs, no significant changes in HR water values were observed.

Overall, the study of the dewatering properties of WP-CNF particulate system via pressure filtration tests was the most effective way to quantify the effect of contact dewatering. Further studies are required for highlighting the direct influence of particle surface area on contact dewatering. Furthermore, the effects of other particle characteristics such as absorptivity, bulk density, compaction, and porosity need to be clearly examined.

## Data Availability

Materials, data and associated protocols are promptly available to readers without undue qualifications in material transfer agreements.
